# Areas of research interest: joining the dots between government and research at last?

**DOI:** 10.12688/f1000research.127542.1

**Published:** 2022-12-13

**Authors:** Kathryn Oliver, Annette Boaz, Giulia Cuccato

**Affiliations:** 1 Public Health Policy, London School of Hygiene & Tropical Medicine, London, UK; 2 Government Office for Science, Department for Business, Energy and Industrial Strategy, London, UK

**Keywords:** evidence use, Areas of Research Interest, policy

## Abstract

**Background:** With the aim of making it easier for researchers to produce policy-relevant research, the UK Government now requires all departments and arms-length bodies to publish annually-updated statements of their evidence needs, called ‘Areas of Research Interest’ (ARIs). We describe how ARIs are produced, and how they are used to support this aim.

**Aims and objectives:** In this paper we offer a description of ARIs and their development by UK governmental departments, and an assessment of how different stakeholders, including academia and funders, have responded to or otherwise used the ARIs.

**Key conclusions:** ARIs are a mechanism for organisations to share their research interests with external audiences in the form of a published document. In addition to this primary aim, they also have a much broader set of uses, including connecting departments with each other and helping intermediaries shape engagement plans. All groups would benefit from more robust evidence to choose effective engagement mechanisms, and more can be done to make the ARIs discoverable and useable. Overall, the ARIs are a useful tool to illuminate, and begin to connect different parts of the research-policy system.

## Introduction: what are areas of research interest?

Although governments, funders and researchers often share a desire for more evidence to support social change, there are significant organisational and procedural barriers in the way. One often-mentioned barrier to evidence use in policy is a lack of policy relevant research ( Armstrong *et al.*, 2006; Oliver *et al.*, 2014; Cairney, 2016; Wye *et al.*, 2019). One way of addressing this barrier is to make it easier for researchers to know about policymakers’ research priorities. In the UK, government departments identify priority research gaps called Areas of Research Interest (ARIs). The Nurse Review into the UK Research Councils (Nurse, 2015) identified a strategic need across government departments to “maintain[] ‘statements of need’, in terms of the most important research questions confronting the Departments” (p.3, Nurse, 2015). By enabling other government departments, funders, and researchers, ARIs were intended to enable policymakers to access relevant evidence and expertise more easily. A similar initiative has been proposed in the US, called the Government Learning Agendas ^1^, where all departments and arms-length bodies are required by the Evidence Act (2019) to publish their knowledge needs.

Many policy and practice organisations including government departments of course communicated their evidence needs before these initiatives (e.g. in Defra’s evidence plan from 2006). However, the ARIs are the first time that every department has been required to publish these publicly, update them annually, and invest resources into their production and use; in effect, 'systematising and embedding the articulation of research interests as part of the fabric of policy making.

## The role of our team

In the UK, the Government Office for Science (GOS) supports the Government Chief Scientific Adviser (GCSA), and holds a cross-government remit to support science capability across departments. It relies on 'soft power' networking and influencing rather than mandating. It also holds the policy for Areas of Research Interest. As part of this remit, Giulia Cuccato led a team of civil servants in the Government Office for Science (GOS) to develop guidance for departments in developing their ARIs, and supporting and tracking their ARI-related activities. Alongside this work, Kathryn Oliver and Annette Boaz have been embedded in GOS to explore and support better production of ARIs, and more effective engagement with them by funders, researchers and intermediaries. We have been involved in supporting the development of ARIs across the UK government, in helping departments use ARIs to access relevant evidence and expertise, and in researching how ARIs could be optimised to support the research-policy system.

Our reflections here are based on our work since 2018 (GC) and 2019 (KO and AB) which has involved working closely with analyst and policy teams in departments across government, and with intermediaries such as the University Policy Engagement Network (UPEN), the National Academies, the What Works Network, and individual universities and researchers, in a practical knowledge mobilisation programme. We draw on these experiences to discuss the strengths and weaknesses of ARIs, and our recommendations to improve their effectiveness as a systems-level intervention.

## Policy outcomes and implications

The ARIs have great potential to enable funders, government, research organisations, researchers, and intermediaries to work together in a more effective way. We have observed that merely producing ARIs is not a sufficient intervention; instead, it requires skilled mobilisation work by people within all these organisations to be able to optimise their production and use. Below, we describe how ARIs are produced, what ARI-related engagement occurs, and how they function as a systems level intervention.

### Embedding production of ARIs across government

When GCSA Patrick Vallance joined GOS in 2018, one of his main goals was to review and increase the scientific capability within government ( Government Office for Science, 2019). This review published recommendations, one of which set out the requirements for annually-updated ARIs to be published by each department. There was considerable variation between government departments. Some departments had existing strong science systems and plans which included an articulation of their research needs, and others had never attempted to map or describe their internal science systems before.

The team with GOS (GC, plus a full-time intern and resources from HEO and SEO level staff) worked with departments to identify the maturity of their science systems, establish their history of developing science plans or equivalents, the connectedness of their science and analysis teams with the policy function within each department, and their consequent ARI support needs. The ARI Fellows (KO and AB) worked with departments offering tailored advice about how to produce and publish ARIs, and how to engage effectively with academics during and after the production process.

As of summer 2022, all departments and arms-length bodies have published at least one version of their ARIs, with at least ten updated in the last two years. Over 1500 ARIs have been published which vary greatly in terms of content, production and associated activities ( Haddon and Sasse, 2018).

### How do departments produce ARIs?

For most departments, the production of ARIs was a primarily internal process. For example, the Departmental Chief Scientist might task the science and analysis team within a department with drafting a set of ARIs. Some departments connected the development of ARIs with their departmental science strategy which set out projected R&D spending for the financial year, and with policy priorities for the department. Some departments engaged with external experts to frame, prioritise and shape their ARIs, for example through their Science Advisory Councils. Others took a highly proactive approach. For instance, Defra held a two-day conference in 2018 with representatives from industry, research and practice to develop their initial ARIs. Once drafted, ARIs are signed off by the relevant directors, the minister, and then published on the UK government website: gov.uk ^2^.

### What are ARIs for?

ARIs were intended to identify strategic research priorities for departments, but in practice we have found that they have a much broader set of uses. We found that departments use them to improve internal working and relationships, to implement the agenda of the Chief Scientist, to support other governmental processes such as spending reviews, the Integrated Review, and the Science Capability review. For some they are a reflection of their policy priorities; for others an articulation of the activities and structures of their internal science system; a statement of likely research commissioning priorities; and/or a statement of research areas around which they would welcome collaboration or input. However, the end products appeared to be mostly appropriate for the departments in question. By and large, they were seen as useful internal tools to negotiate and communicate with policy colleagues around budgets and priorities, and useful external tools to solicit help.

Universities and academics find them useful to plan engagement activities such as workshops and fellowships, but often tend to view them as poorly-written research questions. ARIs can help the research community to understand what government departments want from them. This happens most effectively when there are opportunities for dialogue or a clear narrative about the policy history behind each ARI.

### Addressing ARIs: what engagement methods are best?

Identifying relevant expertise and research is a real challenge for government departments, particularly where resources are limited. Framing problems is an important step for departments, because it dictates what research and which experts are considered relevant and appropriate. We found that officials in government departments were committed to addressing the **challenge of diversity and inclusion in academic-policy engagement**, but we unsure how best to go about improving practice in this area. Departments took different approaches to identifying relevant experts and evidence, ranging from using existing formal structures such as Scientific Advisory Committees and Chief Scientific Advisors, to effectively outsourcing the selection of candidate experts to partners such as the UK Universities Policy Engagement Network. We also found that universities and researchers often reached out directly to departmental officials. While well-intentioned, these approaches often created very significant amount of engagement work for officials on top of their usual engagement activities such as advisory councils. We observed that this had the potential to create competition, duplication and wasted resource on all sides, which is consistent with the literature on academic-policy engagement ( Oliver *et al.*, 2022).

Departments had no clear process to identify which engagement mechanism was the most appropriate for each ARI or set of ARIs. Using a survey of the officials most frequently engaged with ARI-related work, we found over 22 engagement mechanisms were used by departments (see Table 1). Most departments tend to rely on tried and tested approaches (such as commissioning research projects).

**Table 1.** Academic-policy engagement methods used by UK government departments.

Mechanism of Engagement	Departments’ Top 3 Ways to Engage with Academics
Research Projects	11
Advisory Groups/Structures/Events	8
Existing Government Data (Linked Data)	3
Evidence Synthesis	3
Preferred Providers	2
PhD Interns	2
Horizon 2020 and International Research Programmes	2
Fellowships	2
Using Other Gov Scientists	1
SPF	1
Shared Research Cases	1
Shared Outcomes Fund	1
Other Gov Research Resources	1
International Partners	1
Find an Expert	1
Existing Collaborations	1
Co-Financing of Major Surveys	1

We observed that there was scope to broaden the menu of options available to departments when planning and implementing their ARI-related engagement. For example, officials reported primarily using ARIs to commission new research projects or support the generation of new research in other ways. However, we found that there is often – even usually - significant research and expertise already available. In these cases, evidence syntheses or interactive roundtables might be more appropriate and useful methods for gathering relevant research. However, planning a detailed stakeholder engagement plan around ARIs is skilled work which takes resource to plan and execute. For example, skilled staff are needed to support external experts to operate effectively within a policy environment; external experts often require training; time and effort from all side is required to put together a Workplan acceptable to all; and finally, there are operational barriers around political sensitivity, contracts, and finances which take time to resolve.

### Strengths of ARIs: ARIs as a systems intervention

**ARIs work well as an external articulation of research and evidence needs** for departments. They offer funders, intermediaries and researchers’ insights into what departmental research agendas. Universities and intermediaries in particular have used ARIs to develop their own strategic engagement plans (see, e.g. Heckels, 2020). Most departmental ARI documents now contain contact details as well as ‘asks’ and ‘offers’ for each ARI. This makes it easier for funders, intermediaries and researchers to know how to respond (e.g. by getting in touch for a conversation, arranging a research collaboration or responding to a research tender).

**ARIs as a systems intervention**: The ARIs were proposed to encourage the production of more policy-relevant research. This has been described as a ‘deficit model’, suggesting that if decision-makers had better evidence, their decisions would improve. This model has been widely criticised as being based on some fundamentally flawed assumptions about how decision-making works ( Jones and Crow, 2017) and on how evidence informs that process ( Locke, 2002). The ARIs may have been planned to address this illusory ‘deficit’, but in practice perform a far greater range of functions which help to connect the policy research system in complex ways. We have seen the impact of ARIs in:

• Improved understanding of what resources are required, and where, to optimally support ARI work and R&D across government
• Connecting academics with government officials, e.g. Welsh Centre for Public Policy with colleagues in DHSC and DWP; What Works Centre for Cities connecting with government officials across departments
• Influencing new Strategic Priority Fund bids (e.g. NERC SPF programme shaped by the Land Use group to broaden beyond biodiversity to include more social and urban research)
• Enabling discussion between funders and government, e.g. STFC with GOS work on security and resilient; DWP with ESRC grant managers; Innovate UK with GOS colleagues to work on cross-government and UKRI responses to the spending review
• Influencing future funding programmes which follow the themes of our knowledge mobilisation events (e.g. Just transition/vulnerable communities by BA) or drew on our knowledge of ARI mobilisation to inform design (ESRC Policy Fellowship scheme)

The true value of ARIs may be in illuminating the ways in which the research-policy system is connected, and how we can intervene most effectively to support this system.

## Weaknesses of the ARIs: systems challenges

**Not everything can be or is articulated as an ARI:** ARIs are not able to articulate the totality of departmental research needs. For some departments, this is due to political or operational sensitivity, and for others, they prefer to only publish ARIs on topics where they are currently seeking external input. It would be a mistake, therefore, to think of ARIs as a complete and exhaustive list of the topics on which government is seeking input.

**ARIs are not research questions:** Academics frequently describe ARIs as poorly written research questions. An alternative, more useful phrase might be “research needs”, which helps to give the impression that there is a process attached to them, that they are valued, and broader than research questions. They are more usefully thought of as topics for conversation.

**ARIs are difficult to access and analyse:** By 2018, most departments had published at least one version of their ARIs, which then sat on the government website in pdf or html formats. There is as yet no easy way to search for ARIs by topic, department or year, which makes it difficult for researchers to identify relevant topics or potential collaborators This also means that departments are not easily able to identify shared cross departmental areas of interest.

**Finding relevant evidence and expertise takes time and work:** While some departments had resources dedicated to engagement around the ARIs, others did not. While relevant research often exists (as bodies of primary research, in research and practice communities, or in ongoing funding investments), this knowledge is often inaccessible, being behind paywalls or requiring time and skill to find and absorb.

## Actionable recommendations

Areas of Research Interest are a helpful intervention, as a cog in the system which may help us make and use evidence more effectively to inform policy. They are a positive development, without being a silver bullet solution to the knotty problem of evidence production and use. However, much could be done to optimise their production and use.

*Funders* need to support knowledge exchange and evidence synthesis as well as primary research relevant to these areas. At present, funders are often not able to easily access their portfolios of existing investments, which means it is difficult for them to help departments access ARI-relevant existing research or relevant experts. Funders do not appear to be able to track use of ARIs in proposals, and it is not clear how they shape future investments. Finally, funders do not appear to being using ARIs as a basis to identify shared interests) and where they do fund knowledge exchange activities, they do so in an uncoordinated and non-strategic way. Hardly any funders invest in research about evidence production and use, which would produce empirical evidence to guide these activities.

*Intermediaries:* Intermediaries (such as the National Academies, selected What Works Centres such as the Wales Centre for Public Policy) often do have both skilled and resourced individuals who work across academic-policy boundaries. Further investment in brokers and intermediaries would be an effective way to strengthen research-policy engagement. Where there is significant staff turnover in government (as civil servants move roles) Intermediaries can become the repository for organisational memory and could advise departments on their own policy and research engagement history. However, this work is often essentially unpaid, and there is no easy way for the skills and careers of knowledge mobilisers in these organisations to be nurtured. Intermediaries occupy a really important place in the academic policy landscape, enabling them to convene and facilitate cross-field and interdisciplinary discussions. We found that Intermediaries were really valued, able to access experts, bring in diverse voices and knowledge types. However, the brokering organisations we saw (National Academies, What Works Networks, UPEN) did not represent all academics and communities.

*Researchers and universities* need to incentivise effective policy engagement. individual researchers often lack structures to help them understand policy need, so despite the existence of the ARIs, they struggle to address policy agendas. They are not well-trained or incentivised to engage well, and where they do, they tend to actively push experts and expertise out via marketing and PR approaches, rather than thinking about the overall science system. This leads to competition between them for officials’ time, which is inefficient and drives inequity and a lack of diversity in the science advisory system. Training is often about increasing volume and impact of individual researchers or projects. We observed that almost all the university-led projects and engagement activities sought to push university agendas (e.g. institutional profile) or researcher agendas (e.g. individual careers, or importance of particular pieces of evidence). This is not helpful for policy colleagues, who then have to analyse where engagement is most useful and least costly. There is also a growing infrastructure to support knowledge mobilisation within universities, and interestingly across them too. The University Policy Engagement Network is an opportunity to collaborate on and share knowledge exchange training, best practice, and a chance to offer government a more diverse set of experts to draw on.

*Government departments:* need to be clear what is being asked and offered for each ARI. Their current resources and histories of engagement shape their ARI planning, but a broader range of engagement mechanisms would make academic-policy engagement more efficient and effective. Departments may benefit from more support to develop and implement engagement plans effectively, minimising duplication and maximising value for money. There is also a need to track the use of ARIs to understand how they are used and demonstrate to departmental colleagues the value of investing time in ARI development and academic engagement.

All groups would benefit from a more robust evidence base about how to engage most effectively There is considerable scope to improve the process of selecting appropriate next steps, and to expand the range of research-policy engagement mechanisms used. In general, seeking to complement existing structures and processes is most efficient and leads to least disruption.

*Making ARs discoverable:* in their current form (as published ARIs documents from each government department) they can be hard to search and access (especially for cross departmental issues). Making the ARIs accessible would enable researchers and funders to identify trends, themes and topics to inform their future and present work. Government officials would be able to discover areas of shared interest with other departmental colleagues and work on shared priorities together. This would be a great public resource.

*Understanding the impact of ARIs:* The impacts we identified from our ARI-related knowledge mobilisation are not a comprehensive overview of how they are shaping research, engagement and investment activities. It would be useful to have a more exhaustive exploration of who is using ARIs, how, and for what purpose. As we note above, it is unlikely that ARIs will ever be able to capture all governmental research needs or engagement activities. For example, they may be more useful at capturing topics which could inform policy over the medium to long-term than for short term problems.

*Shared problem-framing and priority-setting:* ARIs offer an opportunity for government to think and articulate its strategic aims. To operationalise these aims takes organisational capability which needs to be built over time, both within government and in its external partners. This might be achieved by a cross-government initiative or department taking responsibility for identifying areas of shared interest, a process for prioritisation, and/or a place to optimise engagement between academia and government.

## Conclusions

• By stating their evidence needs, government departments make it easier for researchers to produce or provide relevant research.
• ARIs perform a range of useful functions helping to connect parts of the policy research system in different ways.
• Improving the accessibility of ARIs through a searchable database would increase their utility to all users.
• Government, researchers, funders and intermediaries would benefit from a more robust evidence base about how to engage most effectively to optimise the use of ARIs.

The ARIs are a useful step forward to a more research-informed policy culture. We have highlighted some of the work which is needed to make ARIs useful, and identified points within the system where intervention and support would be beneficial. To make this happen, investment and resource will be required. Overall, these ARIs are an interesting model for other governments wanting to improve research production for and use in policy.

## Author contribution

All authors developed the ideas for the paper and approved the final manuscript.

## Data Availability

All the ARIS are available on https://www.gov.uk/government/collections/areas-of-research-interest . No primary research data was analysed to produce this paper.

## Footnotes

^1^

www.evaluation.gov/evidence-plans/learning-agenda/

^2^

https://www.gov.uk/government/collections/areas-of-research-interest
