# Supplementary material for: Areas of research interest: joining the dots between government and research at last?
Source: F1000Res. 2026 Jan 16;11:1509. Originally published 2022 Dec 13. [Version 2] doi: 10.12688/f1000research.127542.2 (PMC12924004; doi:10.12688/f1000research.127542.2)
Supplement: Peer review history

# Reviewer report: Reviewer response for version 2

**DOI:** 10.5256/f1000research.195197.r456644

**Published:** 2026-04-01

**Authors:** Adam M Wellstead

**Affiliations:** 1 Department of Social Sciences, Michigan Technological University, Houghton, Michigan, USA

This is an interesting paper, but as somebody who had no prior knowledge of APIs, the authors need to better describe exactly what APIs are in the very first paragraph. I needed to double-check by reviewing the government website. https://www.gov.uk/government/collections/areas-of-research-interest

 Perhaps a short description and a link to this website.

 Also, the authors do a nice job describing the process, but it would be helpful if brief examples were included. The authors do this in the "ARIs as a systems intervention." It would provide a nice context for the general reader.

 Minor point: "This activity would beed to be resourced, and collaboration and leadership would need to be incentivised by funders." I assume you meant "need."

 It was an enjoyable read. Good luck!

Does the paper provide a comprehensive overview of the policy and the context of its implementation in a way which is accessible to a general reader?

Partly

Is the discussion on the implications clearly and accurately presented and does it cite the current literature?

Yes

Are the recommendations made clear, balanced, and justified on the basis of the presented arguments?

Yes

Reviewer Expertise:

Public policy

I confirm that I have read this submission and believe that I have an appropriate level of expertise to confirm that it is of an acceptable scientific standard.

# Reviewer report: Reviewer response for version 2

**DOI:** 10.5256/f1000research.195197.r456641

**Published:** 2026-02-20

**Authors:** Martyn Hammersley

**Affiliations:** 1 The Open University, Milton Keynes, England, UK

This revised version is a well-structured and clearly written article that provides an informative account of an intervention in which the authors were involved. This was designed to improve communication between UK Government departments and researchers, and thereby to institute a more effective basis for evidence-based policymaking. The topic is clearly an important one, and the account provided is therefore to be welcomed. I do not see any need for further revisions of the paper.

 However, it does raise several important issues, even if discussion of these was not demanded by the purposes of the article itself. One concerns how evidence from research actually feeds into and shapes departmental decision-making: how does it weigh in relation to other sources of information and other considerations that policymakers must take into account? This is critical for any assessment of the value of ARI-based engagement. A second, connected, issue is about how the changes that the intervention required affected the operation of government departments: were there any negative alongside the positive effects?

 The final issue relates to the other side of the relationship. The authors note that researchers and universities tend to push their own agendas, and they comment that ‘This is not helpful for policy colleagues, who then have to analyse where engagement is most useful and least costly’. This seems to imply that the relationship between the two sides can and should be smooth and direct, and be subordinated to the needs of Government departments. We might ask whether this is possible without research being done in-house rather than by external agents; or, indeed, whether it would be frictionless even then. Is there not always going to be conflict among the different agendas of those involved, especially since there will be some diversity in agenda and schedule on both sides? It is also worth noting that the assumption appears to be that university research, or a significant portion of it, should be directly geared to serving the needs of policymakers (as they define them). That may well be the view from Government, and indeed it reflects a long-term trend towards treating public funding of universities as an investment on which practical ‘returns’ are required. We could ask, though: what is then left of the previously influential notion of a university as an independent academic institution (‘very little’ might be one answer). Furthermore, what are the implications for any belief that we live in a deliberative democracy, which requires funding of research that does not adopt the Government’s policy framework? This is particularly worth pondering given the possibility, in the not-so-distant future, of a government whose commitments are likely to be unpalatable to many academics, so that they will lack any desire to make its policies more effective through being evidence-based.

 As already indicated, I am not suggesting that the authors ought to have addressed these questions in their paper, but they are ones that need to be tackled. And answers to them may point to the need for rather different interventions than that described in the paper.

Does the paper provide a comprehensive overview of the policy and the context of its implementation in a way which is accessible to a general reader?

Yes

Is the discussion on the implications clearly and accurately presented and does it cite the current literature?

Yes

Are the recommendations made clear, balanced, and justified on the basis of the presented arguments?

Yes

Reviewer Expertise:

Research methodology, sociology of knowledge, political sociology

I confirm that I have read this submission and believe that I have an appropriate level of expertise to confirm that it is of an acceptable scientific standard.

# Reviewer report: Reviewer response for version 1

**DOI:** 10.5256/f1000research.140062.r176614

**Published:** 2023-06-16

**Authors:** Sarah Ball

**Affiliations:** 1 School of Social and Political Sciences, The University of Melbourne, Melbourne, Victoria, Australia

This is a very interesting piece, which definitely presents a comprehensive overview of the policy and the context of its implementation in a way which is accessible to a general reader. The aim is descriptive, and it does an excellent job of achieving that aim. The development of ARI's, and the barriers and potential facilitators to their use, is an area which will be of great interest to researchers and practitioners in the UK, but also for other countries looking to facilitate collaboration between government and the research sector. The findings are targeted and actionable, with valuable learnings for funders, intermediaries, governments and researchers. These findings are clear, balanced and well supported by the descriptive research that precedes it.

 The need for more research is also very clear. Given this, I would have appreciated some indication of whether this was part of a larger project which has produced or will be producing research papers/journal articles which step out methodology more clearly. The findings are useful, but I would love to see some more detailed discussion and analysis (while recognising this is not the aim of this paper). Some claims such as 'academics frequently describe ARI's as poorly written research questions' would be worth providing more evidence for, or making more equivocal, in light of this. Without any methodology section, I have some reservations about making claims like that otherwise.  

 My only other note is that there are a few typos in this proof:

- p4 para 5 'we unsure', should be 'were unsure'
- p6 para 4 'collaborators This' missing full stop.
- p6 para 8 ' ) Intermediaries' missing full stop. 
- p6 para 9 'engagement. individual' Individual should be capitalised.
- p7 para 4 'AR's' should (presumably) be ARI's.

Does the paper provide a comprehensive overview of the policy and the context of its implementation in a way which is accessible to a general reader?

Yes

Is the discussion on the implications clearly and accurately presented and does it cite the current literature?

Yes

Are the recommendations made clear, balanced, and justified on the basis of the presented arguments?

Yes

Reviewer Expertise:

Public policy and knowledge translation practices

I confirm that I have read this submission and believe that I have an appropriate level of expertise to confirm that it is of an acceptable scientific standard, however I have significant reservations, as outlined above.

# Response

**Published:** 2026-01-12

**Authors:** Kathryn Oliver

**Affiliations:** London School of Hygiene & Tropical Medicine, UK

This is a very interesting piece, which definitely presents a comprehensive overview of the policy and the context of its implementation in a way which is accessible to a general reader. The aim is descriptive, and it does an excellent job of achieving that aim. The development of ARI's, and the barriers and potential facilitators to their use, is an area which will be of great interest to researchers and practitioners in the UK, but also for other countries looking to facilitate collaboration between government and the research sector. The findings are targeted and actionable, with valuable learnings for funders, intermediaries, governments and researchers. These findings are clear, balanced and well supported by the descriptive research that precedes it.

  

- Many thanks Sarah for your careful review of our paper and apologies for the long delay in responding to your comments which are so helpful and positive.

The need for more research is also very clear. Given this, I would have appreciated some indication of whether this was part of a larger project which has produced or will be producing research papers/journal articles which step out methodology more clearly.

  

- Thanks, this is a good point. We have added more text in the ‘ARI Team and our role’ section to highlight the broader nature of the research undertaken as part of these fellowships, as well as signposting to the other publications out and under way. We have also added more detail about the work we undertook to support the production and use of ARIs, including interviews we did which have informed this paper.

The findings are useful, but I would love to see some more detailed discussion and analysis (while recognising this is not the aim of this paper). Some claims such as 'academics frequently describe ARI's as poorly written research questions' would be worth providing more evidence for, or making more equivocal, in light of this. Without any methodology section, I have some reservations about making claims like that otherwise.  

  

- Yes, take this point. The methods outlined in the ‘roles’ section should help here, but also have been through to make some points more equivocal as you suggest. Appreciate the suggestion.
  My only other note is that there are a few typos in this proof

 

- Many thanks for pointing these out, which have now been corrected.

# Reviewer report: Reviewer response for version 1

**DOI:** 10.5256/f1000research.140062.r158048

**Published:** 2023-02-06

**Authors:** Hannah Durrant

**Affiliations:** 1 Wales Centre for Public Policy, Cardiff University, Cardiff, UK

Thank you for the opportunity to review this valuable and well-written paper. It provides a succinct and much needed assessment of where government areas of research interest (ARIs) have come from in the UK, how they are being produced and used, and their strengths and weaknesses. It does this from a unique combination of insider-researcher perspectives and, in doing so, can claim to practise what it preaches in terms of active and engaged forms of knowledge mobilisation (indeed I think more can be made of this). Actionable recommendations are provided for all stakeholders – many stress the importance of intermediary mechanisms for knowledge mobilisation to bring government evidence needs together with experts and expertise to understand what evidence exists and what new knowledge could be produced to inform decision-making. They raise the urgent need for more robust evidence on what mechanisms for knowledge mobilisation are most effective, when, under what conditions, and why.

 I have a few comments and suggestions for areas to strengthen the paper further, which are outlined below. There are also a few, small typographical points which may be picked up in a robust copy edit, e.g., some words missing from the sentence in the first paragraph that starts “ *By enabling other government departments, funders, and researchers*…”; there is a quotation mark in front of the word “ *systematising*..” in paragraph 2; at the bottom of page 5, it should be ‘Wales Centre…’ not " *Welsh Centre*…"; top of page 6, " *BA*" should be 'AB'; middle of page 6, it should be “funders do not appear to be using…” rather than “ *being using*…”; etc. These are small and infrequent but would perfect the paper.

**Comments and suggestions**

 The paper would benefit from elaborating a bit more (e.g., within the section on the role of the team) on the ARI fellowship design, and the specific activities undertaken that generated the insight that underpins the claims made later in the paper (e.g., in the middle of page 4 about what universities and academics find useful about ARIs, how UPEN functions as an intermediary, and points about competition and duplication arising from direct engagement).

1. It would be worth making it clear for a broad base of readers that two of the authors (KO and AB) were/are embedded researchers.
2. It would also be very valuable to provide a brief explanation of embedded research and any assessment of evaluation as one among many mechanisms to enhance knowledge mobilisation. Given that it is a highly relational and engaged approach to brokering knowledge to support decision-making, it seems a highly appropriate approach to choose, particularly in light of limitations raised about efforts to merely ‘push’ research evidence out to policy. That said, the approach is not without difficulties (some of which may be captured in points made about the transactional costs of engagement and the lack of support and recognition for these roles and approaches). Is there an opportunity to reflect on this approach in the context of discussion about the benefits and challenges of mechanisms for knowledge mobilisation in response to government ARIs? The Wye *et al.* (2019) paper already cited could provide a starting point for conceptualising embedded research/ers. More importantly, it would be valuable to outline how the team designed their approach to maximise the potential of their fellowships to achieve the aims and avoid any pitfalls.
3. I think it would be useful to provide a little more detail on the nature of the work of the team with government department analysts and policy teams, as well as with intermediaries, universities and researchers, in order to underpin the perspectives on ARIs that you report in the section on what ARIs are for. What kinds of interactions happened? How often? What was the purpose? Were there any limitations that should be taken into account?

You note variation between government departments in the production of ARIs, differences in what they are intended for, and that not all government ‘evidence needs’ are/can be articulated as ARIs. These observations suggest implications for whether ARIs actually signal evidence need on the *most important* departmental research questions, as intended in the Nurse Review (N.B. could you provide the reference for the Nurse Review, 2015?). Even if we accept a flawed ‘deficit model’ that misrepresents policymaking processes (your critique here is very valid), it remains unlikely that evidence can inform decisions if the most important evidence needs are not articulated. It may be worth reflecting on this, alongside other points made about whether ARIs are likely to generate/stimulate the mobilisation of the most relevant and useful evidence for addressing policy priorities and to meet the stated ultimate aspiration for evidence to inform social change. This may strengthen the point that ARIs should be seen as providing a starting point for focused conversation and that their impact (and limits) under these conditions need to be better understood in order to inform a multi-method approach (including methods more likely to get to the most important policy research questions) for strengthening research-policy engagement on important topics of government priority that ARIs don’t cover.

 In the section on 'Strengths of ARIs: ARIs as a systems intervention', I wondered if the team had observed any impacts of ARIs in terms of enabling university-based intermediaries (e.g., policy engagement support within individual universities or within UPEN) to focus the ways they seek to coordinate and connect academics to government officials? I do not think the Wales Centre for Public Policy (WCPP) provides a good example of how ARIs work to connect academics with government officials. This is not core to WCPP's work or a typical activity for the Centre, but good examples are useful and may be found elsewhere.

 Finally, given that the paper stresses the importance of intermediaries and knowledge mobilisers/brokers (albeit underpinned by an urgently needed evidence base on what works), I think the conclusions would be strengthened by adding a point on actively supporting knowledge mobilisation around ARIs, which requires action and investment by funders, universities, intermediaries and government. The buy-in of these stakeholders may be crucial for plugging the evidence gap on effective mechanisms for better research-policy engagement.

 I think these comments are minor and overall this paper makes an important contribution to better understanding engagement with government ARIs. Thank you again for an enjoyable read.

Does the paper provide a comprehensive overview of the policy and the context of its implementation in a way which is accessible to a general reader?

Yes

Is the discussion on the implications clearly and accurately presented and does it cite the current literature?

Partly

Are the recommendations made clear, balanced, and justified on the basis of the presented arguments?

Yes

Reviewer Expertise:

Evidence use in policymaking; knowledge mobilisation; policy design and evaluation; loneliness and community wellbeing.

I confirm that I have read this submission and believe that I have an appropriate level of expertise to confirm that it is of an acceptable scientific standard, however I have significant reservations, as outlined above.

# Response

**Published:** 2026-01-12

**Authors:** Kathryn Oliver

**Affiliations:** London School of Hygiene & Tropical Medicine, UK

Responses:

  

- Thanks so much for your careful reading of our paper and apologies for the long delay in  addressing these very helpful pointers.

I have a few comments and suggestions for areas to strengthen the paper further, which are outlined below. There are also a few, small typographical points which may be picked up in a robust copy edit, e.g., some words missing from the sentence in the first paragraph that starts “ *By enabling other government departments, funders, and researchers*…”; there is a quotation mark in front of the word “ *systematising*..” in paragraph 2; at the bottom of page 5, it should be ‘Wales Centre…’ not " *Welsh Centre*…"; top of page 6, " *BA*" should be 'AB'; middle of page 6, it should be “funders do not appear to be using…” rather than “ *being using*…”; etc. These are small and infrequent but would perfect the paper.

  

- Thanks, a careful proof read has been undertaken. Appreciate you taking the time to point these errors out.
**Comments and suggestions**

 

1. The paper would benefit from elaborating a bit more (e.g., within the section on the role of the team) on the ARI fellowship design, and the specific activities undertaken that generated the insight that underpins the claims made later in the paper (e.g., in the middle of page 4 about what universities and academics find useful about ARIs, how UPEN functions as an intermediary, and points about competition and duplication arising from direct engagement).

- Good suggestion, this has been done.

1. It would be worth making it clear for a broad base of readers that two of the authors (KO and AB) were/are embedded researchers.

- Good suggestion, clarified

1. It would also be very valuable to provide a brief explanation of embedded research and any assessment of evaluation as one among many mechanisms to enhance knowledge mobilisation. Given that it is a highly relational and engaged approach to brokering knowledge to support decision-making, it seems a highly appropriate approach to choose, particularly in light of limitations raised about efforts to merely ‘push’ research evidence out to policy. That said, the approach is not without difficulties (some of which may be captured in points made about the transactional costs of engagement and the lack of support and recognition for these roles and approaches). Is there an opportunity to reflect on this approach in the context of discussion about the benefits and challenges of mechanisms for knowledge mobilisation in response to government ARIs? The Wye *et al.* (2019) paper already cited could provide a starting point for conceptualising embedded research/ers. More importantly, it would be valuable to outline how the team designed their approach to maximise the potential of their fellowships to achieve the aims and avoid any pitfalls.

- Thanks for this suggestion, which gave me much food for thought. I agree the Fellow model deployed in our case was somewhat unusual as being both practical / delivery focused, and offering the opportunity for research and reflection about the model itself, with both elements built explicitly into the design of the fellowship. We have added some sentences in the ‘role of our team’ and discussion section to clarify this; however, a broader discussion is probably outside the scope of this paper which aimed to describe the production and use of ARIs for a wider audience. We would like to return to the question of the importance of embeddedness in more detail in a further paper – thanks for the suggestion.

1. I think it would be useful to provide a little more detail on the nature of the work of the team with government department analysts and policy teams, as well as with intermediaries, universities and researchers, in order to underpin the perspectives on ARIs that you report in the section on what ARIs are for. What kinds of interactions happened? How often? What was the purpose? Were there any limitations that should be taken into account?

- Thanks, this has been added in the ‘how do departments produce ARIs’ sections.

1. You note variation between government departments in the production of ARIs, differences in what they are intended for, and that not all government ‘evidence needs’ are/can be articulated as ARIs. These observations suggest implications for whether ARIs actually signal evidence need on the *most important *departmental research questions, as intended in the Nurse Review Even if we accept a flawed ‘deficit model’ that misrepresents policymaking processes (your critique here is very valid), it remains unlikely that evidence can inform decisions if the most important evidence needs are not articulated. It may be worth reflecting on this, alongside other points made about whether ARIs are likely to generate/stimulate the mobilisation of the most relevant and useful evidence for addressing policy priorities and to meet the stated ultimate aspiration for evidence to inform social change. This may strengthen the point that ARIs should be seen as providing a starting point for focused conversation and that their impact (and limits) under these conditions need to be better understood in order to inform a multi-method approach (including methods more likely to get to the most important policy research questions) for strengthening research-policy engagement on important topics of government priority that ARIs don’t cover.

- Thanks, great points and addressed in the discussion and actionable recommendations.

1. (N.B. could you provide the reference for the Nurse Review, 2015?).

- Apols, added now

 

1. In the section on 'Strengths of ARIs: ARIs as a systems intervention', I wondered if the team had observed any impacts of ARIs in terms of enabling university-based intermediaries (e.g., policy engagement support within individual universities or within UPEN) to focus the ways they seek to coordinate and connect academics to government officials? I do not think the Wales Centre for Public Policy (WCPP) provides a good example of how ARIs work to connect academics with government officials. This is not core to WCPP's work or a typical activity for the Centre, but good examples are useful and may be found elsewhere.

- Thanks, we had been referring to a specific discussion we set up between WCPP academics and government; reworded to make this clearer. Again, we think there are now many more examples to draw out which we will return to in a later paper.

8. Finally, given that the paper stresses the importance of intermediaries and knowledge mobilisers/brokers (albeit underpinned by an urgently needed evidence base on what works), I think the conclusions would be strengthened by adding a point on actively supporting knowledge mobilisation around ARIs, which requires action and investment by funders, universities, intermediaries and government. The buy-in of these stakeholders may be crucial for plugging the evidence gap on effective mechanisms for better research-policy engagement.

  

- A great point -thanks, added to the recs.
